# Comparison of Two *Ginkgo biloba* L. Extracts on Oxidative Stress and Inflammation Markers in Human Endothelial Cells

**DOI:** 10.1155/2019/6173893

**Published:** 2019-06-25

**Authors:** Stefano Piazza, Barbara Pacchetti, Marco Fumagalli, Fabrizia Bonacina, Mario Dell'Agli, Enrico Sangiovanni

**Affiliations:** ^1^Department of Pharmacological and Biomolecular Sciences, Università degli Studi di Milano, Milano, Italy; ^2^Linnea SA, Riazzino, Switzerland

## Abstract

Atherosclerosis is characterized by interaction between immune and vascular endothelial cells which is mediated by adhesion molecules occurring on the surface of the vascular endothelium leading to massive release of proinflammatory mediators. *Ginkgo biloba* L. (Ginkgoaceae) standardized extracts showing beneficial effects are commonly prepared by solvent extraction, and acetone is used according to the European Pharmacopoeia recommendations; the well-known *Ginkgo biloba* acetone extract EGb761® is the most clinically investigated. However, in some countries, the allowed amount of solvent is limited to ethanol, thus implying that the usage of a standardized *Ginkgo biloba* ethanol extract may be preferred in all those cases, such as for food supplements. The present paper investigates if ethanol and acetone extracts, with comparable standardization, may be considered comparable in terms of biological activity, focusing on the radical scavenging and anti-inflammatory activities. Both the extracts showed high inhibition of TNF*α*-induced VCAM-1 release (41.1-43.9 *μ*g/mL), which was partly due to the NF-*κ*B pathway impairment. Besides ROS decrease, cAMP increase following treatment with ginkgo extracts was addressed and proposed as further molecular mechanism responsible for the inhibition of endothelial E-selectin. No statistical difference was observed between the extracts. The present study demonstrates for the first time that ethanol and acetone extracts show comparable biological activities in human endothelial cell, thus providing new insights into the usage of ethanol extracts in those countries where restrictions in amount of acetone are present.

## 1. Introduction

Atherosclerosis is characterized by a massive chronic inflammation with increased oxidative stress which in turn induces the adhesion of immune cells (i.e., monocytes, leucocytes) to the vascular endothelium and their subsequent migration into the vessel wall. The interaction between immune and vascular endothelial cells is mediated by vascular adhesion molecule-1 (VCAM-1), intercellular adhesion molecule-1 (ICAM-1), and E-selectin occurring on the surface of the vascular endothelium.

In humans, the expression of adhesion molecules has been consistently observed in atherosclerotic plaques [1–3], where high levels of soluble adhesion molecules are considered useful risk predictors of cardiovascular events in healthy populations [4]. Their expression can be transcriptionally regulated by inflammatory cytokines such as tumor necrosis factor-*α* (TNF-*α*), which stimulate adhesion molecules mRNA and cell surface expression through the activation of NF-*κ*B and AP-1 pathways [5]. These transcription factors are redox-sensitive and strongly activated by proinflammatory and prooxidant mediators, including reactive oxygen species (ROS).

The expression of adhesion molecules occurring in several inflammatory models, including TNF-*α*-induced endothelial inflammation, is modulated by the second messenger cyclic AMP (cAMP) as well [6–8]. Notably, molecules able to increase cAMP levels, such as the phosphodiesterase inhibitors (i.e., theophylline), are able to impair leucocyte adhesion in vascular cells [7, 9–11]. Nowadays, botanicals exhibiting anti-inflammatory mechanisms through cAMP increase and NF-*κ*B inhibition, other than antioxidant activity in endothelial cells, are investigated to counteract the development of vascular diseases.

*Ginkgo biloba* L. is an ancient medicinal plant which has been used in traditional Chinese medicine for thousands of years. Although both leaves and seeds are currently used as herbal medicine in China, leaves are considered the unique source of active principles in many countries, and standardized extracts from leaves are used for supplying pharmaceutical formulations or as ingredient of food supplements.

Phytochemicals occurring in *Ginkgo biloba* L. leaves include biflavones, terpene trilactones (ginkgolides A, B, C, J, P, and Q and bilobalide), flavonol glycosides, proanthocyanidins, and other polyphenols [12, 13].

*Ginkgo biloba* leaf extracts show a variety of biological activities, mostly against cardiovascular [14] and neurological diseases, including regulating effects on the entire vascular system of veins, arteries, and capillaries. *Ginkgo biloba* extracts stimulate lipolysis [15], promote metabolism of brain cells and sensory neurons, and are widely used to treat memory loss, protect against ROS, and improve mild cognitive impairment and cerebrovascular insufficiency [16–19].

Standardized ginkgo extracts should contain flavonoids (22.0-27.0%), expressed as flavonol glycosides, and 5.0-7.0% of terpene lactones including ginkgolides A, B, and C and bilobalide [20], and the daily dose in adults and elderly should be 240 mg according to EMA monograph [21].

Studies in the literature clearly demonstrate that an adequate standardization of the extracts is mandatory for efficacy, and *Ginkgo biloba* standardized extracts represent the pharmaceutically used formulations recommended to achieve beneficial effects.

*Ginkgo biloba* L. standardized extracts are commonly prepared by solvent extraction such as water, methanol, ethanol, or acetone [22]. European Pharmacopoeia recommends extraction of *Ginkgo* powder with acetone twice for 30 minutes [23]. The clinically investigated extract EGb761® is an acetone *Ginkgo biloba* extract [24, 25].

In some countries (i.e., Japan), the amount of acetone allowed in food supplements is limited to ethanol, thus implying that the usage of standardized ethanol extracts is mandatory [26]. However, to the best of our knowledge, no studies have compared the anti-inflammatory and antioxidant effects of acetone and ethanol extracts in human endothelial cells, which are considered one of the preferred biological targets of *Ginkgo biloba* extracts.

The aim of the present study was (1) to chemically profile aqueous acetone vs. aqueous ethanol extracts from *Ginkgo biloba* leaves similarly standardized to identify differences among components; (2) to investigate if both the extracts may be considered comparable as anti-inflammatory and antioxidant agents; and (3) to elucidate the mode of action underlying the effect observed *in vitro*.

## 2. Materials and Methods

### 2.1. Plant Material and Extraction

G4E (*Ginkgo biloba* aqueous ethanol extract) and G24 (*Ginkgo biloba* aqueous acetone extract, EPG246) were prepared by Linnea SA (Riazzino, CH) (https://www.linnea.ch/) and provided, as powder, for biological assays. Ginkgo leaves were collected by plantations cultivated in Europe and North America under controlled conditions, according to [27].

For the preparation of *Ginkgo biloba* G4E, ginkgo leaves were submitted to solid/liquid extraction using aqueous ethanol as extraction solvent. The extracted fractions were then concentrated under reduced pressure to remove the extraction solvent and purified by filtration on resin column and liquid/liquid extractions. For the preparation of G24, *Ginkgo biloba* leaves were submitted to solid/liquid extraction using aqueous acetone as extraction solvent. The extracted fractions are then concentrated under reduced pressure to remove the extraction solvent and purified by liquid/liquid extractions. In both cases, organic solvent was then replaced with water and the aqueous phase was finally concentrated and dried to obtain the final powdered extract.

Patented standardized extraction and purification processes were applied to obtain extracts with consistent composition in pharmacologically active compounds.

### 2.2. HPLC Method for Determination of Flavonoids and Terpenes

Flavonoids and terpenes occurring in G24 and G4E were determined according to EP current edition. Briefly, for flavonoid detection, the extracts (200 mg) were dissolved in methanol (20 mL). 15.0 mL of diluted hydrochloric acid and 5 mL of water were added and diluted to 50 mL with methanol. 10 mL of the previous solution was hydrolyzed in a 100°C water bath for 25 minutes. An aliquot (10 *μ*L) of solution was injected: detector: spectrophotometer at 370 nm; column size: l = 0.125 m, diameter = 4 mm; stationary phase: C18 (5 *μ*m); temperature: 25°C; mobile phase: A: 0.3 g/L of phosphoric acid R adjusted at pH = 2.0; mobile phase B: methanol; gradient elution: time 0 (min) (A : B 60 : 40), time 1 (A : B 60 : 40), time 20 (A : B 45 : 55), time 21 (A : B 0 : 100), time 25 (A : B 0 : 100); flow rate: 1 mL/min. Quercetin dehydrate was used as reference compound. For terpene determination, the extract (120 mg) was dissolved in phosphate buffer solution (10 mL, pH 5.8) by stirring. Solution was transferred into a column containing 15 g of kieselguhr. Column was eluted with ethyl acetate (100 mL). Solvent was evaporated and the residue dissolved in 2.5 mL of the mobile phase. An aliquot (100 *μ*L) of solution was injected: detector: refractometer at 35°C; column size: l = 0.25 m, diameter = 4 mm; stationary phase: C8 (5 *μ*m); temperature: 25°C; mobile phase: tetrahydrofuran : methanol : water (10 : 20 : 75, *v*/*v*); flow rate: 1 mL/min. Method was applied according to the European Pharmacopoeia.

### 2.3. Cell Culture

Human endothelial cells (HUVECs, CRL-1730, ATCC, Virginia, USA), which are immortalized cells with life expectancy of 50-60 doublings according to ATCC specification, were grown at 37° in Ham's F-12 Kaighn's Medium (Ham F12K) (Sigma-Aldrich, Milano, Italy) supplemented with 100 U penicillin/mL, 100 mg streptomycin/mL, 2 mM glutamine, and 20% heat-inactivated foetal calf serum (FCS) (Euroclone S.p.A, Pero, Italy), in a humidified atmosphere containing 5% CO_2_. Cells (passage number 2-30) were treated with the indicated concentration of *Ginkgo biloba* L. extracts or vehicle alone (<0.2% DMSO). Curcumin 10 *μ*M was used as reference compound.

### 2.4. Cytotoxicity

The cytotoxicity of the extracts was evaluated by the 3,4,5-dimethylthiazol-2-yl-5-diphenyltetrazolium bromide (MTT) assay, as previously described [28].

### 2.5. Measurement of Soluble Adhesion Molecules

For the measurement of soluble ICAM-1, VCAM-1, and E-selectin adhesion molecules, HUVECs were grown in 24-well plates (20,000 cells/well) for 72 h; cells were treated with the proinflammatory stimulus (TNF*α* at 20 ng/mL) and the extracts under study for 6 h. Afterwards, the medium was removed and stored at -20°C until the assays. Soluble ICAM-1, VCAM-1, and E-selectin were quantified in the media by an enzyme-linked immunosorbent assay (ELISA). ICAM-1 was detected by Human ICAM-1 ABTS ELISA development kit (PeproTech, Rocky Hill, NJ, USA) and E-selectin by RayBio Human E-Selectin ELISA (RayBiotech, Norcross, USA), respectively, by measuring in duplicate 200 and 100 *μ*L of culture medium and following each manufacturer's instruction. For VCAM-1, Corning 96-well EIA/RIA plates were coated with the rabbit anti-human VCAM-1 (100 pg/well) (PeproTech, USA) overnight at 4°C. After blocking with BSA, 250 *μ*L of samples was transferred into wells at room temperature for 2 h. The amount of VCAM-1 in the samples was detected by using biotinylated rabbit anti-human VCAM-1 antibody (50 pg/well) and streptavidin-HRP-conjugated protein (PeproTech, USA). The 3,3′,5,5′-tetramethylbenzidine (TMB) substrate reaction was measured by spectrophotometry (signal read: 450 nm, 0.1 s), and human VCAM-1 standard curve, ranging from 0 to 10 pg/mL, was used to quantify VCAM-1 in the samples. Curcumin (10 *μ*M) was used as reference compound.

### 2.6. Measurement of Cell Surface Adhesion Molecules by Flow Cytometry

HUVECs were plated in 6-well plates (60,000/well) and treated after 48 hours with TNF*α* (20 ng/mL) in the presence of *Ginkgo biloba* extracts (G4E and G24) for 6 hours. Controls were treated only with the vehicle (≤0.2%). Cells were then washed once with PBS, harvested, and spun at 2000×g for 5 minutes. Cells were stained in 50 *μ*L of PBS with 2% BSA (Sigma-Aldrich) in the presence of the following antibodies: PE-conjugated Mouse anti-Human CD106 (VCAM-1) (BD Pharmingen cod. 555647), APC-conjugated Mouse anti-Human CD54 (ICAM-1) (BD Pharmingen cod. 559771), and PE-Cy-conjugated Mouse anti-Human CD62E (E-SEL) (BD Pharmingen cod. 550040). Cells were incubated with antibodies for 30 minutes at 4°C in the dark, then washed once in PBS with 2% BSA (Sigma-Aldrich), spun at 2000×g, and resuspended in 250 *μ*L of PBS/2% BSA. Cells were acquired with NovoCyte flow cytometer (ACEA Biosciences, San Diego, CA, USA) and analyzed by NovoExpress software (ACEA Biosciences).

### 2.7. Measurement of Gene Expression by qRT-PCR

For mRNA analysis, HUVECs were seeded in 100 mm plates (5 × 10^5^ cells/10 mL) and treated with TNF*α* (20 ng/mL) for 6 hours in the presence of *Ginkgo biloba* extracts (G4E and G24); mRNA was extracted with RNeasy Mini Kit (Qiagen, Milano, Italy) and quantified by spectrophotometric analysis at 260 nm (NanoDrop ND-100 spectrophotometer, Euroclone, Italy).

Real-time PCR reactions were performed on 10 ng mRNA/well in the Real-Time System Bio-Rad CFX384 (Bio-Rad, CA, USA) using an iTaq™ Universal SYBR Green One-Step Kit (Bio-Rad), which allowed mRNA reverse transcription and cDNA labelling. Amplification of the gene coding for VCAM-1, ICAM-1, or E-selectin (E-SEL) (Primmbiotech, Cambridge, UK) was assessed. The thermal cycling protocol required a preliminary step for reverse transcription at 50°C for 10 min, followed by polymerase activation step (95°C, 5 min) and 40 cycles with 95°C denaturation step for 10 s and annealing/extension at 60°C for 30 s. All the samples were tested in triplicate, and the relative expression of genes was calculated by normalizing the threshold cycle (Ct) of each gene with the Ct of GAPDH mRNA to correct for variation in RNA loading. The effect of the extracts on 84 inflammatory cytokines and receptor genes was evaluated by RT^2^ Profiler PCR Array PAHS-011Z (Qiagen, Milano, Italy).

### 2.8. Measurement of ROS Production

To evaluate the effect of the extracts on ROS formation, HUVECs were grown in black 96-well plates (10,000 cells/well) for 72 hours and then treated with the extracts for 24 hours. After the treatment, cells were incubated for 1 h with a fluorescent probe (CM-H2DCFDA, Invitrogen by Thermo Fisher Scientific, USA) and then washed with PBS 1X before the prooxidant stimulation with H_2_O_2_ (500 *μ*M for 1 h). The fluorescence of the internalized probe was read, after a final wash with PBS, by a microplate reader at 535 nm (Wallac Envision, Perkin Elmer, Waltham MA, USA). Trolox (500 *μ*M) or curcumin (10 *μ*M) was used as reference compounds.

### 2.9. NF-*κ*B Nuclear Translocation

To assess the effect of the extracts on the NF-*κ*B (p65) nuclear translocation, HUVECs were plated in 100 mm plates at the density of 5 × 10^5^ cells. After 72 h, cells were treated for 1 h with the proinflammatory mediator and the extracts under study. Nuclear extracts were prepared using Nuclear Extraction Kit from Cayman Chemical Company (Michigan, USA) and stored at -80°C until assayed. The same amount of total nuclear proteins (10 *μ*g/well), quantified by the Bradford method (Bio-Rad), was loaded into the wells. NF-*κ*B translocation was measured by spectroscopy at 450 nm, 0.1 s (VictorX3, Perkin Elmer, Waltham MA, USA). Data were expressed considering as 100% the absorbance related to the cytokine-induced NF-*κ*B translocation. The results were the means of at least three experiments performed in duplicate. EGCG 20 *μ*M was used as reference compound.

### 2.10. I*κ*B*α* Phosphorylation

The effect of G4E and G24 on I*κ*B*α* phosphorylation was evaluated by IstantOne™ ELISA (Life Technologies, Carlsbad, USA), using antibodies against both phosphorylated and unphosphorylated forms of I*κ*B*α*. Cells were seeded in 12-well plates (60,000 cells/well) and incubated with the proteasome inhibitor MG132 5 *μ*M (Sigma-Aldrich) for 2 hours, then TNF*α* (20 ng/mL) and ginkgo extracts (G4E and G24) were added to cells for 5 min; medium was removed and cells lysed for 10 min at RT with 200 *μ*L/well of Cell Lysis Mix. Sample lysate was quantified for total protein content by Bradford assay. ELISA was performed in triplicate on 10 *μ*g of proteins/well, reading the absorbance at 450 nm (VictorX3, Perkin Elmer, Waltham MA, USA). Phospho-I*κ*B*α* measurement was normalized with the absorbance of the unphosphorylated protein.

### 2.11. Measurement of Cyclic AMP

HUVECs cultured in 6-well plates and treated 48 hours late (60,000/well) were lysed with 0.1 M hydrochloric acid at RT for 20 min. Cells were collected using a scraper and subjected to centrifugation at 1000 × g for 10 min. The supernatant was transferred into new tubes and total protein content was evaluated by the Bradford method. Samples normalized on the total protein content were diluted 1 : 2 with assay buffer and directly used for evaluation of intracellular cAMP level by cyclic AMP ELISA kit (Cayman Chemical Company, cod. 581001, Ann Arbor, MI, USA) according to the manufacturer's protocol. Absorbance was measured at a wavelength of 405 nm using an ELISA microplate reader (VictorX3, Perkin Elmer, Waltham MA, USA).

### 2.12. Statistical Analysis

All data are expressed as mean ± s.d. of at least three experiments. Data were analyzed by unpaired one-way analysis of variance (ANOVA) followed by Bonferroni test by post hoc test. Statistical analyses were done using GraphPad Prism 6.0 software (GraphPad Software Inc., San Diego, CA, USA). *p* < 0.05 was considered statistically significant. IC_50_s were calculated using the GraphPad Prism 6.0 software.

## 3. Results and Discussion

### 3.1. Characterization of *Ginkgo biloba* Extracts

**Figure 1:**
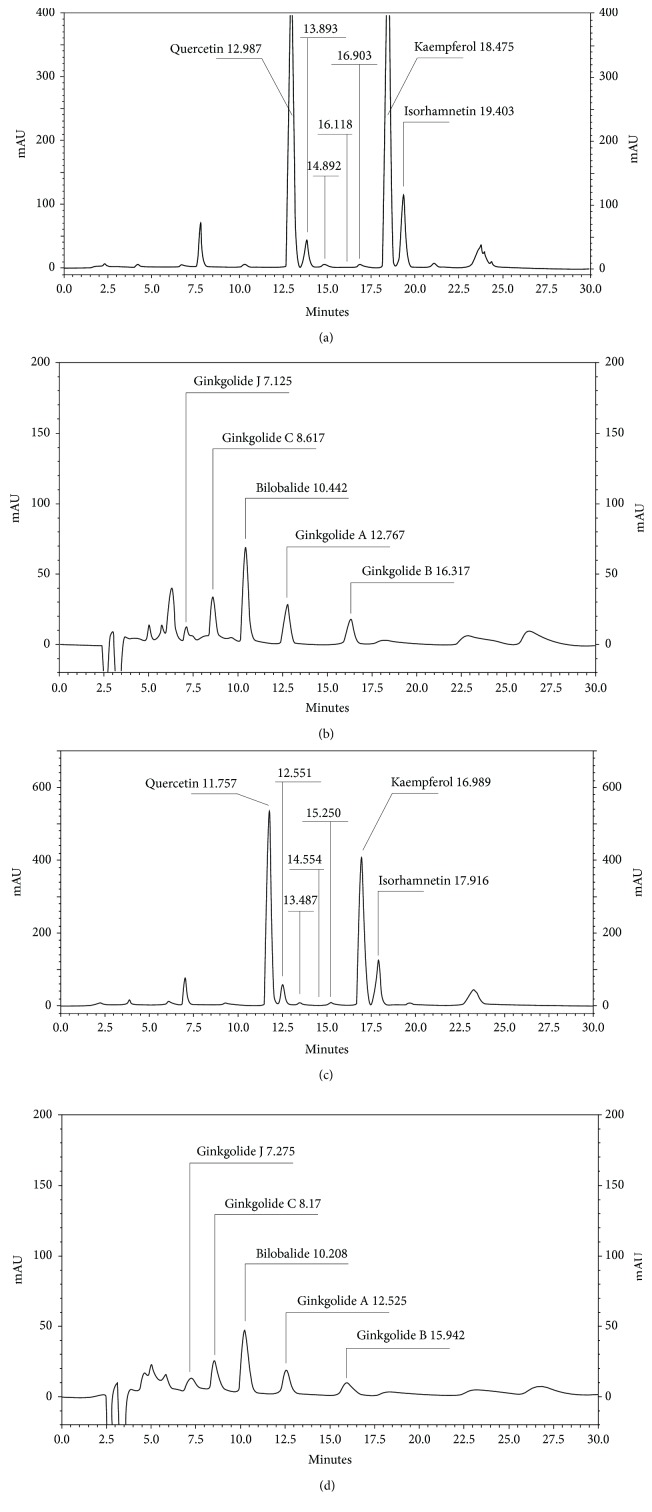
HPLC characterization of G4E and G24 extracts. Flavonoids and terpenes occurring in G24 and G4E were determined according to EP current edition. (a) Flavonoids in G24 extract; (b) ginkgolides and bilobalide in G24 extract; (c) flavonoids in G4E extract; (d) ginkgolides and bilobalide in G4E extract. G24: acetone extract; G4E: ethanol extract.

Figure 1 reports HPLC profiles of G4E and G24. The extracts showed comparable profiles in flavonoid analysis, showing peaks corresponding to quercetin, kaempferol, and isorhamnetin. Similarly, terpene analysis identified peaks corresponding to ginkgolides (A, B, J, and C) and bilobalide in both the extracts.

Table 1 reports characterization of both G4E and G24 extracts. 20 different batches of each extract were analyzed for total content of flavonol glycosides, proanthocyanidins, and bilobalide. The extracts showed the same profile in terms of proanthocyanidins, bilobalide, and flavonoid content. According to these results, the major classes of constituents occurring in *Ginkgo bilob*a acetone extract, which is considered the most suitable to provide biological activity, are also present in the ethanol G4E extract at comparable concentrations. Standardization was applied as follows: 24% flavone glycosides and 6.0% terpene lactones for both G24 and G4E.

**Figure 2:**
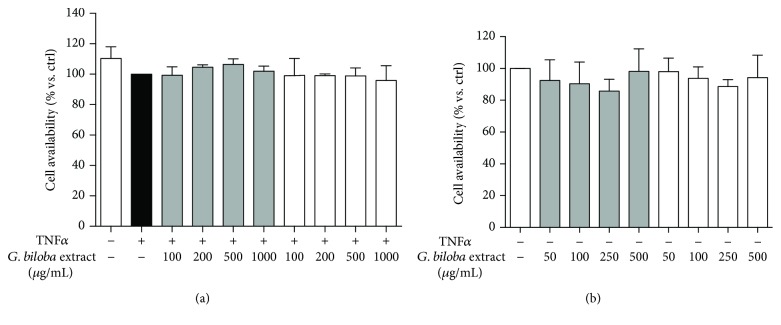
Ginkgo extracts did not impair HUVEC viability in the experimental conditions. Cytotoxicity was neither exhibited during treatment with extracts and TNF*α* for 6 h (a) or treatment with extracts for 24 h (b).

Thus, we performed a panel of experiments to investigate if similar profiles parallel comparable anti-inflammatory and antioxidant activity in human endothelial cells. No cytotoxicity at concentrations used in the present study was observed in endothelial cells (Figure 2).

### 3.2. Ginkgo Extracts Inhibit Cell Surface and Adhesion Molecule Release in Human Endothelial Cells

**Figure 3:**
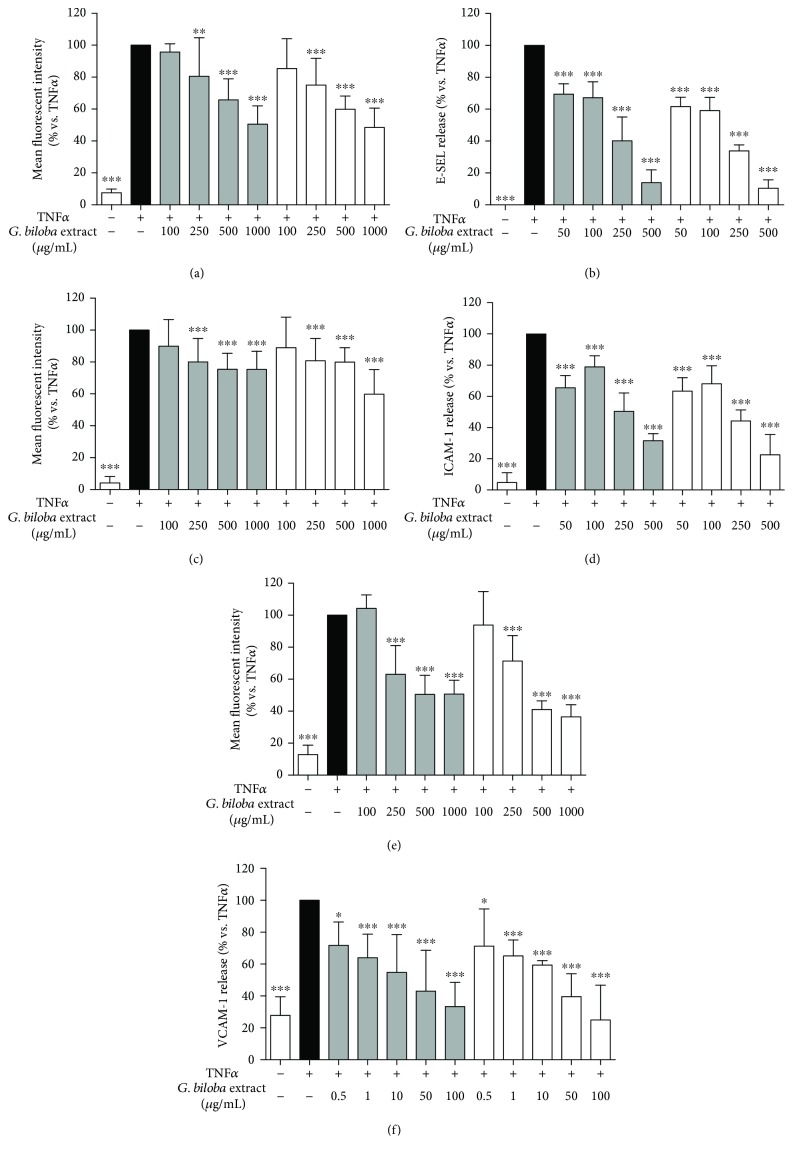
Ginkgo extracts inhibit soluble and cell surface adhesion molecules in human endothelial cells. G4E (grey bars) and G24 (white bars) similarly inhibited E-selectin (a) and VCAM-1 (e) cell surface expression in human endothelial cells; a slight statistically significant inhibition (20-30%) was observed on ICAM-1 (c) at concentrations starting from 250 *μ*g/mL. The inhibition of the extracts on E-selectin (b), ICAM-1 (d), and VCAM-1 (f) release was more pronounced. Curcumin 10 *μ*M was used as reference inhibitor of adhesion molecules in all the experiments (average inhibition of 77% for E-selectin, 96% for ICAM-1, and 72% for VCAM-1).

In the first set of experiments, the ability of the extracts to impair soluble and cell surface VCAM-1, ICAM-1, and E-selectin in human endothelial cells was investigated. Results are shown in Figure 3. Both the extracts inhibited in a concentration-dependent way adhesion molecule release induced by TNF*α*; however, the extracts slightly inhibited membrane ICAM-1 starting from 250 *μ*g/mL (Figure 3, Table 2). Both the extracts showed higher activity in inhibiting soluble than the membrane forms. Ginkgo extracts showed higher activity on soluble VCAM-1 (41.1 and 43.9 *μ*g/mL for G4E and G24 extracts, respectively) whereas the inhibitory effect on ICAM-1 and E-selectin was less pronounced (Table 2 and Figure 3). Our findings agree with previous investigations by Chen et al., who demonstrated inhibition of adhesion molecules by a *Ginkgo biloba* standardized extract provided by Dr. Schwabe [5], which is considered comparable to G24 extract.

### 3.3. Ginkgo Extracts Inhibit NF-*κ*B Pathway in Human Endothelial Cells

Proinflammatory cytokines, such as TNF*α*, activate NF-*κ*B pathway leading to expression of a variety of proinflammatory mediators, including adhesion molecules. Thus, NF-*κ*B activation represents a key regulator of adhesion molecule expression in human endothelial cells. NF-*κ*B is also known as a redox-sensitive transcription factor activated by ROS [29], thus showing proinflammatory and prooxidant properties. NF-*κ*B activation mainly occurs via I*κ*B kinase- (IKK-) mediated phosphorylation of I*κ*B*α* [30]. Thus, inhibitors of I*κ*B*α* phosphorylation may impair the NF-*κ*B pathway leading to an anti-inflammatory effect.

**Figure 4:**
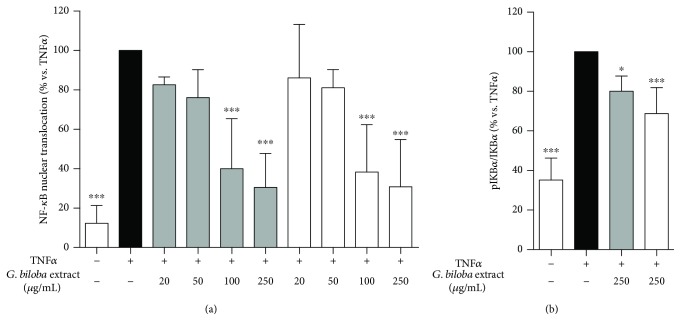
Ginkgo extracts inhibit NF-*κ*B pathway in human endothelial cells. G4E (grey bars) and G24 (white bars) inhibited the NF-*κ*B nuclear translocation induced by TNF*α* in a concentration-dependent fashion (a). Epigallocatechin-3-O-gallate (EGCG) 20 *μ*M was used as reference compound (51% inhibition). At the highest concentration tested (250 *μ*g/mL), G4E and G24 decrease phosphorylation of the NF-*κ*B inhibitor I*κ*B*α* (b). G24: acetone extract; G4E: ethanol extract.

To evaluate if the extracts could impair NF-*κ*B activation, HUVECs were treated with G4E and G24 for 1 hour; then, nuclear translocation from the cytoplasm into the nucleus was measured by ELISA, as previously described in Materials and Methods. Both the extracts inhibited NF-*κ*B translocation in a concentration-dependent fashion (Figure 4(a)), with comparable IC_50_ (75.8 and 79.9 *μ*g/mL for G4E and G24, respectively). Moreover, the extracts showed a statistically significant decrease of I*κ*B*α* phosphorylation at 250 *μ*g/mL, leading to a reduced proteasomal degradation of the NF-*κ*B inhibitor (Figure 4(b)). Interestingly, we observed a significant effect on cell surface expression of adhesion molecules starting from the same concentration (250 *μ*g/mL) thus remarking NF-*κ*B impairment as the main mode of action of the extracts. Both inhibition of the NF-*κ*B nuclear translocation and I*κ*B*α* phosphorylation was confirmed by western blot assays (data not shown).

Previous papers reported the effect of *Ginkgo biloba* extracts as inhibitors of the NF-*κ*B activation in a variety of cell lines including endothelial cells [5, 31–33]. Some important beneficial effects of EGb761 extract, which is comparable to G24 for extraction procedure and standardization, are due to the NF-*κ*B impairment [31]. Our results clearly show that both G4E and G24 extracts similarly inhibit NF-*κ*B translocation at low concentrations, with the same IC_50_.

### 3.4. Ginkgo Extracts Inhibit ROS Production in Human Endothelial Cells

During inflammation, immune cells release high amount of ROS which induce oxidative stress in endothelial cells. It is widely known that moderate amounts of ROS have beneficial effects in human health contributing to fight invasion of pathogens or promoting tissue repair processes. Moreover, uncontrolled generation of ROS leads to reduced levels of endogenous antioxidant enzymes (superoxide dismutase, glutathione peroxidase, and catalase), causing oxidative tissue damage.

To investigate if the extracts may counteract oxidative stress showing antioxidant properties, HUVECs were treated with G4E and G24 for 24 hours; thus, cells were incubated with a fluorescent probe for one hour. Finally, a typical prooxidant stimulus (H_2_O_2_, 500 *μ*M) was added for 1 hour to increase ROS production in HUVEC.

**Figure 5:**
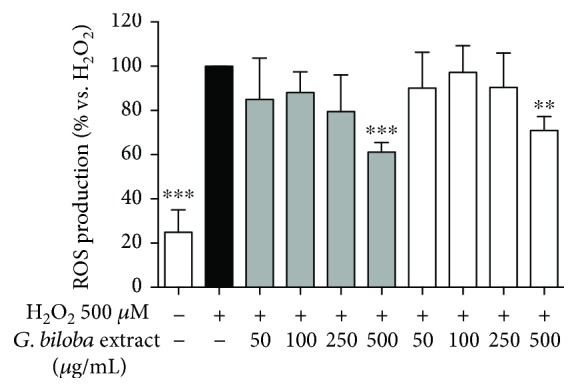
Ginkgo extracts slightly affect ROS production in human endothelial cells. Both the extracts show slight effects on ROS formation, with significant inhibition at the highest concentration (500 *μ*g/mL). G4E (grey bars) and G24 (white bars). Trolox 500 *μ*M was used as reference compound (100% inhibition).

Both the extracts inhibited ROS production, although the effect was significant only at the highest concentration tested (Figure 5). Our data agree with previous papers reporting reduction of ROS production and oxidative stress although in different *Ginkgo biloba* extracts and cell models [5, 34, 35]. However, this is the first report demonstrating that the inhibitory effect on ROS formation in human endothelial cells by G4E and G24 is comparable.

### 3.5. Effect of Ginkgo Extracts on Adhesion Molecule Gene Expression in Human Endothelial Cells

**Figure 6:**
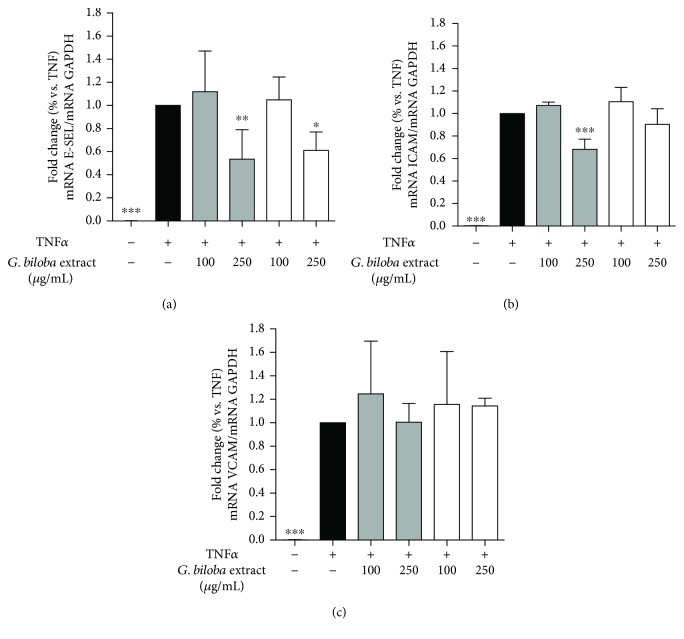
Effect of ginkgo extracts on adhesion molecules at transcriptional level in human endothelial cells. Ginkgo extracts exhibited a similar inhibitory effect on transcription of adhesion molecules: at 250 *μ*g/mL, G4E (grey bars) and G24 (white bars) showed 50% and 40% inhibition of E-selectin transcription, respectively (a), while VCAM-1 mRNA was not impaired (c); the slight inhibition of ICAM-1 transcription was significant only for G4E extract (30% inhibition) (b).

Since G4E and G24 inhibited soluble and cell surface expression of adhesion molecules, we assayed mRNA analysis at the same experimental conditions to verify the effect at transcriptional level. Interestingly, at 250 *μ*g/mL, extracts were able to inhibit E-selectin (40-50%) and slightly ICAM-1 (10-30%), whereas VCAM-1 transcription was not affected (Figure 6) thus suggesting that E-selectin impairment is regulated by different mechanisms with respect to ICAM-1 and VCAM-1.

Collins et al. [36] collected in detail evidences on how these proteins are regulated at transcriptional level in endothelial cells: NF-*κ*B/I*κ*B*α* system plays a crucial role in TNF-*α*-induced expression of adhesion molecules, but a relatively small number of other transcription factors must assemble with NF-*κ*B to generate unique transcriptional-activating complexes. Focusing on E-selectin transcription, ATF/CRE (*cyclic AMP responsive element*) cooperates with NF-*κ*B, thus E-selectin promoter is susceptible to cAMP regulation. On the other hand, several experiments in HUVECs demonstrated that an increase in cAMP levels caused by cAMP analogues or phosphodiesterase type 3 and 4 inhibitors leads to the inhibition of E-selectin and VCAM-1, but not ICAM-1 membrane expression [7, 10]. On the contrary, ICAM-1 (both mRNA and surface expression) is upregulated by persistent cAMP elevation during TNF*α*-induced endothelial inflammation, thus suggesting antiphosphodiesterase activity as a possible antagonistic mechanism for extracts displaying NF-*κ*B and ICAM-1 inhibition [37].

### 3.6. Effect of Ginkgo Extracts on Inflammatory Cytokine and Receptor Gene Expression in Human Endothelial Cells

Since G4E and G24 inhibited the NF-*κ*B pathway in our cell model, we evaluated the overall ability of the extracts to impair a panel of genes involved in endothelial inflammation; data are shown in the supplementary material. TNF*α* significantly overexpressed 25 genes out of 84, among them 10 were expressed in a statistically significant way; however, both the extracts did not decrease the corresponding mRNA levels, except for the member of the TNF family TNFSF10, which was slightly reduced by acetonic extract G24 (Figure S1).

### 3.7. Ginkgo Extracts Exhibit cAMP-Elevating Activity

To assess the involvement of cAMP in the anti-inflammatory effect observed in HUVECs, the extracts were tested for their ability to increase cAMP levels in comparison with the well-known phosphodiesterase inhibitor theophylline 500 *μ*M at 1 and 6 hours.

**Figure 7:**
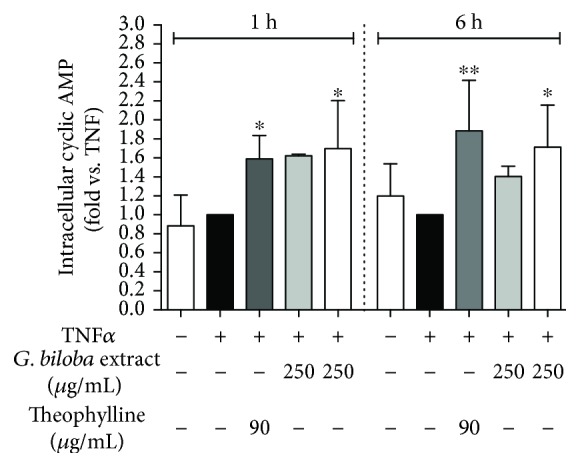
Ginkgo extracts increase intracellular cAMP level in human endothelial cells. G4E (grey bars) and G24 (white bars) 250 *μ*g/mL increased intracellular cyclic AMP in HUVECs after 1 and 6 h treatments. The effect was comparable to the well-known phosphodiesterase inhibitor theophylline 500 *μ*M (90 *μ*g/mL), which increased intracellular cyclic AMP of 1.59- and 1.89-folds, respectively, after 1 h and 6 h, compared to TNF*α*.

**Figure 8:**
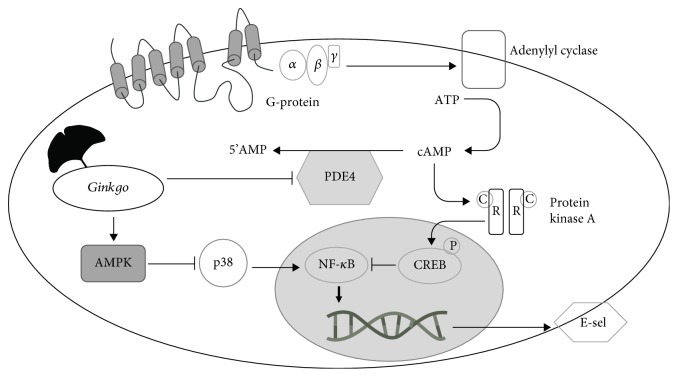
Proposed mechanism to explain the effect of ginkgo extracts on signaling cascade involved in E-selectin regulation. *Ginkgo* could reduce the NF-*κ*B activation by increasing intracellular cAMP levels acting on PDE4 or by enhancing AMPK activity. Lines with arrows indicate activation, while lines with bars indicate inhibition.

Both G4E and G24 (250 *μ*g/mL) increased cAMP levels after 1 and 6 hours (Figure 7) although the effect was statistically significant only for G24. Our results seem to corroborate the hypothesis that cAMP may negatively regulate E-selectin transcription/surface expression and VCAM-1 surface expression acting on the NF-*κ*B system. On the contrary, cAMP elevation may have an opposite effect on ICAM-1 inhibition. Remarkably, the increase of cAMP following 6 hours of treatment with G24 or G4E paralleled the loss of inhibitory effect on ICAM-1 transcription. Indeed, our findings are partially confirmed by other authors demonstrating that EGB761 extract preferentially inhibits PDE type 4 in HUVECs [38]; in this study performed in primary HUVECs, the extract was effective at lower concentrations thus suggesting that immortalized endothelial cells used in the present study may be less sensitive to the pharmacological effects of the extract. Moreover, it has been previously demonstrated that ginkgolide C (10-500 *μ*M) is able to increase cAMP and cGMP in platelets [39]. Our results, in addition to those occurring in the literature, suggest for the first time that inhibition of E-selectin by *Ginkgo biloba* extracts in HUVECs may be due, at least in part, to cAMP elevation resulted by PDE inhibition; indeed, it has been previously reported that ginkgo constituents are inhibitors of cGMP- and cAMP-dependent phosphodiesterase [40] [41]. In addition, ginkgo leaf extracts may impair NF-*κ*B through AMPK activation [32]. It has been demonstrated that other phosphodiesterase inhibitors, such as cilostazol, activate AMPK pathway, which may lead to the impairment of proinflammatory adhesion molecules in platelets and endothelial cells (Figure 8) [42, 43].

Concentrations used in the present research may be considered high and not easily reachable in vivo; however, it has been previously reported that ginkgolides and bilobalide are able to be absorbed in vivo, reaching significant concentrations in blood, according to EMA monograph [21]. Bioavailability of ginkgo flavonoids including kaempferol and quercetin is still controversial. Although variability exists within different formulations, it has been reported that ginkgo flavonoids are absorbed and reach the plasma and brain in detectable amounts in vivo whereas other authors detect only the corresponding glucuronides [44–47]. Taken together, these results allow to hypothesize that in addition to lactones, flavonoids or their metabolites could efficiently show biological activity in endothelial cells [13]. In addition, synergistic effects among ginkgo active compounds cannot be excluded.

## 4. Conclusions

European Pharmacopoeia recommends extraction of *Ginkgo* powder with acetone; our findings demonstrate for the first time that ethanol (G4E) and acetone (G24) extracts show comparable anti-inflammatory and antioxidant activity in human endothelial cells. In some countries, including Japan, solvents allowed in food supplements or plant extracts are limited to ethanol, thus implying that the usage of standardized ethanol extracts is mandatory. Thus, the results reported herein show that ginkgo extracts made from extraction with acetone or ethanol exhibit similar anti-inflammatory and antioxidant activities, providing new insights into the usage of ethanol extracts in those countries where restrictions in amount of acetone are present. However, different effects of the extracts on other pathways cannot be excluded.

This work clarifies the mode of action by which ginkgo extracts inhibit adhesion molecules acting on different steps of the NF-*κ*B pathway increasing cAMP levels in human endothelial cells.

Concentrations of the extracts used in the present work may be reached in vivo since lactones, particularly ginkgolides, are adsorbed and reach blood at significant concentrations thus contributing to the biological effects reported herein.

## Figures and Tables

**Table 1:** Characterization of bilobalide, proanthocyanidins, and flavonoids in ginkgo extracts.

	G24 extract	G4E extract
	% (mean ± s.d.)	
Bilobalide	2.94 ± 0.13	2.87 ± 0.13
Proanthocyanidins	3.62 ± 2.65	4.81 ± 2.42
Flavonol glycosides	25.31 ± 1.04	25.03 ± 0.94

Results are the mean ± s.d. of 20 different batches of G24 (acetone) or G4E (ethanol) extracts.

**Table 2:** IC_50_ of ginkgo extracts on adhesion molecules and NF-*κ*B translocation.

Form	Membrane	Soluble	Membrane	Soluble	Membrane	Soluble	Nuclear
	VCAM-1	sVCAM-1	ICAM-1	sICAM-1	E-sel.	sE-sel.	NF-*κ*B translocation
G4E	476.1 ± 103.7	1.5 ± 2.4	>1000	226.7 ± 27.4	849.1 ± 169.0	159.2 ± 22.7	75.8 ± 19.9
G24	372.4 ± 39.1	1.6 ± 1.6	>1000	152.5 ± 26.8	726.8 ± 160.3	115.9 ± 15.5	79.9 ± 18.3

IC_50_s (*μ*g/mL) were calculated using GraphPad Prism 6.0 software (GraphPad Software Inc., San Diego, CA, USA). Data represent the mean of at least three experiments performed in duplicate or triplicate. G24: acetone extract; G4E: ethanol extract.

## Data Availability

The data used to support the findings of the manuscript 6173893 entitled “Comparison of Two Ginkgo biloba L. Extracts on Oxidative Stress and Inflammation Markers in Human Endothelial Cells” are included within the article or in the supplementary information file(s).

## Supplementary Materials

Supplementary Materials The heat map on the left shows 25 genes which are positively regulated by TNF*α* treatment in HUVECs, while the effect of G4E (grey bars) and G24 (white bars), on the genes which are significantly overexpressed by TNF*α*, is shown in the respective histograms. The extracts did not significantly impair the expression of the inflammatory genes in HUVECs, with the exception of G24 for TNFSF10 gene.
