# Supplementary material for: Comparison of Two Ginkgo biloba L. Extracts on Oxidative Stress and Inflammation Markers in Human Endothelial Cells
Source: Mediators Inflamm. 2019 Jun 25;2019:6173893. doi: 10.1155/2019/6173893 (PMC6614955; doi:10.1155/2019/6173893)

Figure S1

The heat-map on the left show 25 genes which are positively regulated by TNFα treatment in HUVEC cells, while the effect of G4E (grey bars) and G24 (white bars), on the genes which are significantly overexpressed by TNFα, is shown in the respective histograms. The extracts did not significantly impair the expression of the inflammatory genes in HUVEC cells, with the exception of G24 for TNFSF10 gene.

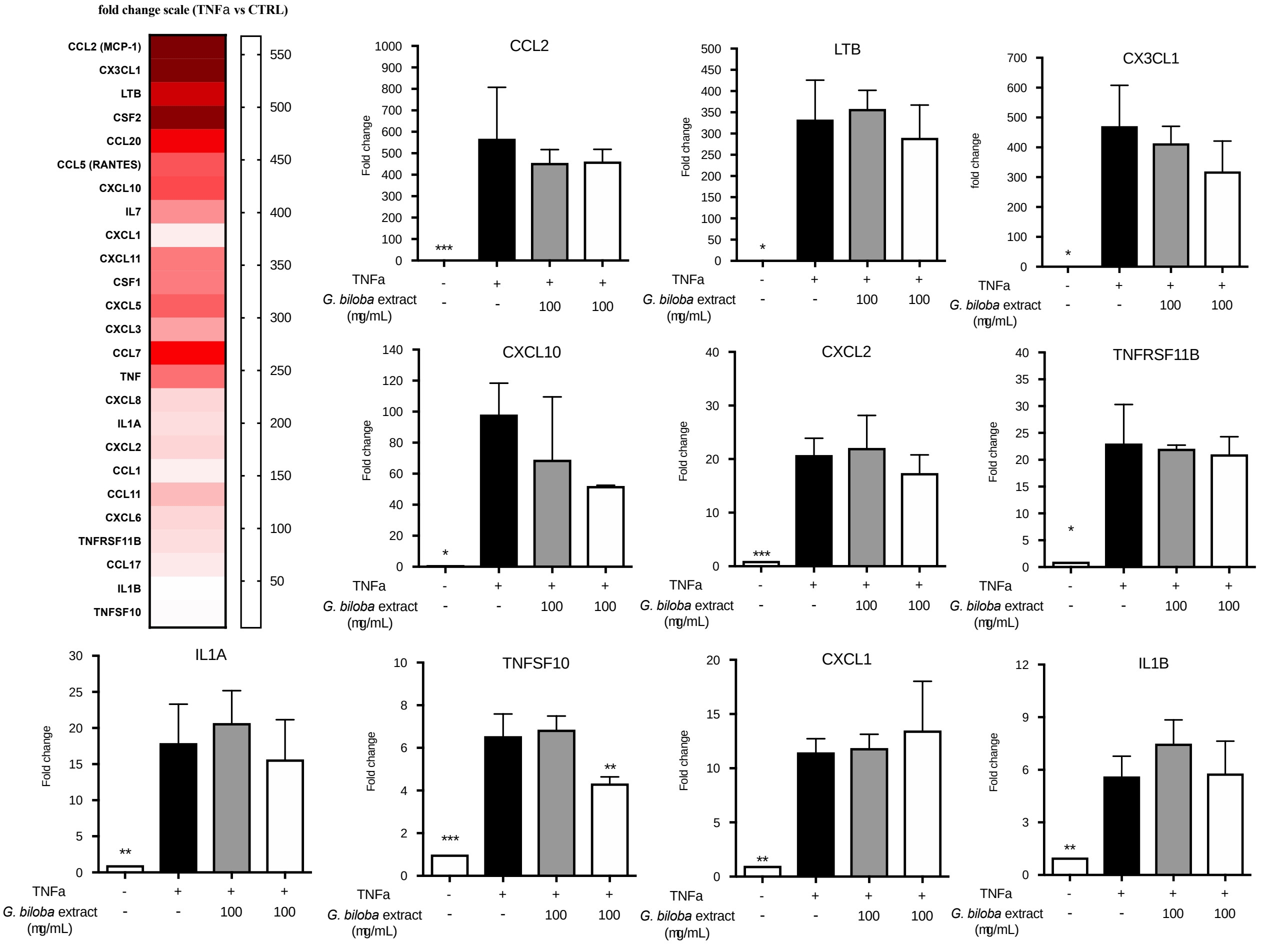

Supplement: Supplementary Materials — The heat map on the left shows 25 genes which are positively regulated by TNFα treatment in HUVECs, while the effect of G4E (grey bars) and G24 (white bars), on the genes which are significantly overexpressed by TNFα, is shown in the respective histograms. The extracts did not significantly impair the expression of the inflammatory genes in HUVECs, with the exception of G24 for TNFSF10 gene. [file 6173893.f1.pdf]
